#  “His Lyre Is Now Attuned Only to Woe”

**DOI:** 10.3201/eid1405.052008

**Published:** 2008-05

**Authors:** Polyxeni Potter

**Affiliations:** * Centers for Disease Control and Prevention, Atlanta, Georgia, USA

**Keywords:** Giovanni Battista Tiepolo, Giambattista Tiepolo, Bust of an Old Man, Venetian painting, fresco painting, art and science, influenza, the elderly, immunocompromised patients, Petrarch, emerging infectious diseases, about the cover

**Figure:**
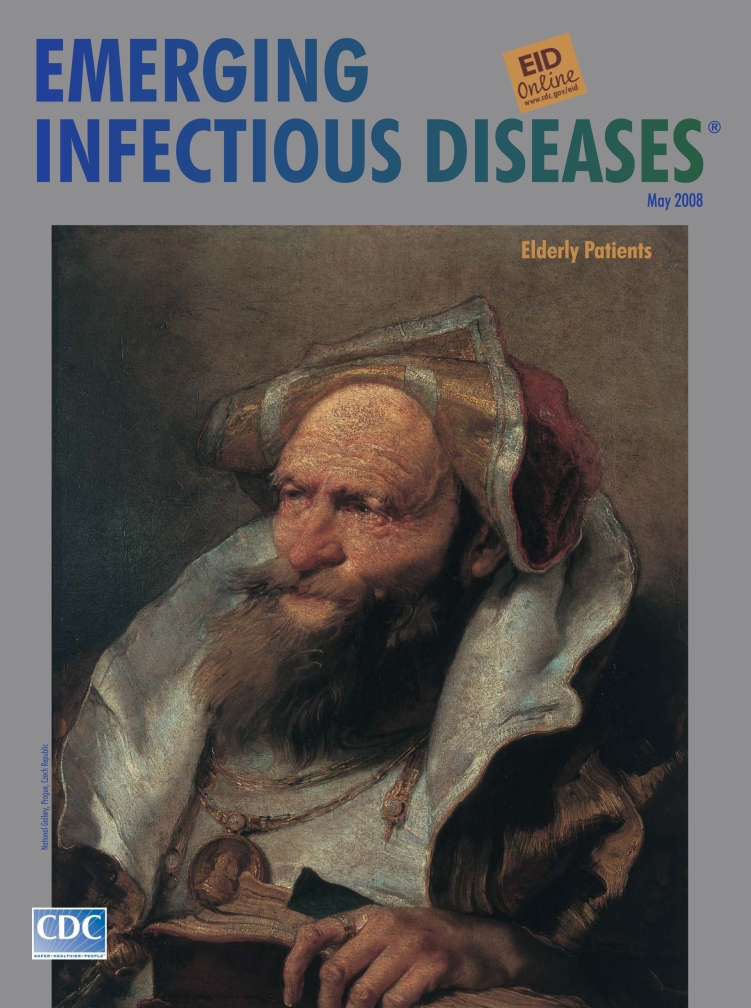
**Giovanni Battista (Giambattista) Tiepolo (1696–1770) Bust of an Old Man (c. 1751–1755).** Oil on canvas (61 cm × 50.5 cm). National Gallery, Prague, Czech Republic

—Petrarch

“Dreams and fables I fashion,” wrote 18th-century Italian poet and librettist Pietro Metastasio, “and even while I sketch elaborate fables and dreams upon paper ... I so enter into them that I weep and am offended at ills I invented” (*1*). Metastasio might have been describing the work of his contemporary Giambattista Tiepolo, whose art blended history, mythology, legend, and scripture in a grand manner. His frescoes, which graced the palaces and princely courts of Europe, embodied the notion popular in his day that painting, like theater and opera, was staged fiction and should engage the viewer on an imaginary level (*2*).

Tiepolo was born in Venice, itself a fanciful and theatrical city of canals, lagoons, piazzas, and palaces, the center of artistic splendor, even during its decline in the 18th century. From a family of painters, draftsmen, and etchers, he was apprenticed as a youth to academic master Gregorio Lazzarini. He married Maria Cecilia, sister of painters Gianantonio and Francesco Guardi, and by age 21, he was an established painter too. He had nine children. Two followed in their father’s footsteps and became his assistants; one, Domenico, became a great artist in his own right (*3*).

Tiepolo was influenced by the work of 16th-century master Paolo Veronese, particularly in his use of sumptuous often anachronistic costumes, and was often called *Veronese redivivus* (a new Veronese) (*4*). He learned eloquence and drama from Titian and Tintoretto and admired the work of his contemporary Giovanni Battista Piazzetta. His education was complex and varied, enriched by his circle of friends, patrons, collectors, and connoisseurs of the stage and its extravagance, among them cosmopolitan courtier and art critic Francesco Algarotti, who wrote a treatise on opera.

Extremely versatile, Tiepolo mastered multiple artistic forms and media (etchings, watercolors, oils) and made brilliant use of chiaroscuro―use of light and dark to create depth. He produced altarpieces as well as portraits and was a prodigious sketcher who infused his work with humor and irony. He is most acclaimed for his frescoes in the Palazzo Labia in Venice, the Royal Palace in Madrid, and the Würzburg Residenz in Germany.

The fresco, a style of painting on wet plaster requiring “rapid and resolute” execution, suited Tiepolo’s talent and the sense of immediacy central to his work. His mastery of composition, color, and perspective enabled him to make the most of available spaces. He either ignored architectural boundaries or used them to frame or expand images and create convincing illusions in large biblical and mythologic scenes. He often collaborated with renowned architect Girolamo Mengozzi-Colonna, who designed for the theater, to increase illusionist effects by incorporating architectural features for decorative richness, affirming a centuries-old tradition of exchange between stage design and painted narrative.

“All spirit and fire” was how Tiepolo was described in his time, also the time of François Boucher, Jean-Honoré Fragonard, Jean-Antoine Watteau, and Canaletto (*5*). Though praised as “the most celebrated of the virtuosi,” he remained approachable and accommodating (*5*). “Painters should aim to succeed in great works,” he believed, “the kind that can please noble, rich people, for it is they who determine the fortunes of the Masters, and not other people, who cannot buy paintings of great value” (*6*).

The most representative artist of the period, Tiepolo bridged the drama and grandeur of the Baroque and the frivolity and decorativeness of Rococo. In the last years of his life, the rise of neoclassicism damaged his career but did not have a long lasting impact on his legacy.

“The mind of the painter must always be directed towards the Sublime, the Heroic, towards perfection,” Tiepolo believed (*6*). He valued the world of heroes and gods and imagination over appearances. His taste for perfection also showed in his drawings of archeological artifacts: busts, statues, and decorative heads, particularly distinguished by their emphasis on exotic costumes, turbans, and antique features. His interest extended to bust-length depictions of philosophers or sages. These drawings were props for large paintings, sometimes previewed by collectors and patrons.

Bust of an Old Man, on this month’s cover, likely belongs to these independent pieces within Tiepolo’s work, which were later transferred into prints by his son. They were produced in the 1750s as studies but soon became valued by collectors as original works. Apart from their skillful expression of physiognomy, these portraits also displayed the artist’s facility with light effects on unusual fabrics, inspired by Dutch painting (*7*).

“Help me to crease the pleats of an emerald sleeve Giambattista Tiepolo, Paolo Veronese,” mused poet Derek Walcott in “Tiepolo’s Hound,” expressing the intensity and complexity of feeling evoked by the artist’s work (*8*). Tiepolo’s old man is spectacularly attired in shimmering silk and velvet tossed flamboyantly over his head and shoulders, adorned with a large pendant. His ringed hand grasps a book. His face is alert and focused, eyes glaring, mouth protruding deliberately, beard untamed.

Old age is not a frequent subject in art. This study by Tiepolo is in line with his choice of noble figures, in this case the holder of knowledge and experience as he might appear on life’s stage in some elaborate production with a huge cast. Even as the old man recedes into his finery, he shows nothing of weakness, dependency, or illness. This venerable icon exemplifies verisimilitude as practiced by Tiepolo and proposed by Goethe as the object of all art, “Not to counterfeit nature but to create a harmonious whole that gave a semblance of reality” (*2*).

The sage as envisioned by Tiepolo, Rembrandt, and other Old Masters is endangered in our times. By definition immunocompromised, he and all the elderly are at increased risk for multiple health threats to their viability and prowess. In this journal issue alone, increases are noted in hospitalizations for pneumonia and community-acquired staphylococcal infections (*9*). The persistence of Osler’s “old man’s friend” indicates that attention has been scant to the problems of the elderly. Yet they are us. As Petrarch put it, “Men go abroad to admire the heights of mountains, the mighty waves of the sea, the broad tides of rivers, the compass of the ocean, and the circuits of the stars, yet pass over the mystery of themselves without a thought” (*10*).
